# The Promoting Effects of Fermented Bile Acid on Growth Performance and Intestinal Health of *Litopenaeus vannamei* Through the Modulation of Lipid Metabolism and Gut Microbiota

**DOI:** 10.1155/anu/2064288

**Published:** 2025-02-06

**Authors:** Qing Guo, Haili Ma, Lu Zhao, Wenwen Liu, Houfa Zhao, Zuyue Liu, Cuimin Mu, Xuepeng Wang

**Affiliations:** ^1^Shandong Provincial Key Laboratory of Zoonoses, Key Laboratory of Efficient Utilization of Non-Grain Feed Resources (Co-Construction by Ministry and Province) of Ministry of Agriculture and Rural Affairs, Shandong Agricultural University, Taian 271018, China; ^2^Anhui Chem-Bright Bioengineering Co., Ltd., Huaibei 235025, China; ^3^Jining Leyuhui Ecological Agriculture Development Co., Ltd., Jining 272000, China

**Keywords:** bile acids, growth performance, intestinal microbiota, metabolite, white shrimp

## Abstract

Two types of bile acids (BAs), named fermented bile acids (FBAs) and 170HDa, were produced by a biological approach to assess their effects on growth performance, metabolism, and intestinal health in white shrimp. In this study, five experimental diets were prepared with varying levels of FBAs (0.02% for A1, 0.03% for A2, 0.04% for A3, 0.05% for A4, and 0.06% for A5) and five diets containing different concentrations of 170HDa (0.02% for B1, 0.03% for B2, 0.04% for B3, 0.05% for B4, and 0.06% for B5). Additionally, positive diets (P) with commercial BAs at a level of 0.04%, along with and negative diet (N) without BA supplementation, were included as controls. FBAs and 170HDa were found to improve the growth performance including final weight, weight gain, and specific growth rate and reduce the activities of aspartate aminotransferase and alanine aminotransferase in hemolymph. The alkaline phosphatase (AKP) activity of hemolymph in shrimp treated with FBAs was generally higher than in groups treated with 170HDa and the control groups. However, the acid phosphatase (ACP) activity of hemolymph in shrimp treated with FBAs showed similar levels to those treated with 170HDa and the control groups. The gene expression levels of antilipopolysaccharride factor (ALF) and proPO were significantly lower in most FBAs and 170HDa-treated groups compared to the control groups (*p* < 0.05). Additionally, the gene expression levels of α2M in hepatopancreas were significantly higher in the 170HDa-treated groups compared to those in the FBAs-treated group (*p* < 0.05). The FBAs and 170HDa significantly enhanced the intestinal health by boosting the proinflammatory capacity and increasing the diversity of the intestinal microbiota, thereby combating pathogenic microorganisms. Notably, there was a significant increase (*p* < 0.05) in the abundance of Proteobacteria, Firmicutes, Tenericutes, and Cyanobacteria at the phylum level, as well as *Vibrio*, *Rhodobacter*, and *Shewanella* at the genus level, respectively. These findings indicate that dietary FBAs and 170HDa have positive effects on growth performance and intestinal health by modulating lipid metabolic profiles, immune responses, the integrity of intestinal wall, and the diversity of intestinal microbes in white shrimp. This study suggests that FBAs and 170HDa could serve as effective dietary supplements to enhance shrimp production and health management in aquaculture, providing a promising strategy for sustainable aquaculture practices.

## 1. Introduction

Lipid, as a vital nutrient, serves as a crucial energy source in the diet of shrimp and play an important role in providing the fatty acids necessary for their growth and survival [1, 2]. In addition to supplying sufficient energy, lipids are important carriers of certain vitamins and serve as synthetic precursors for various vitamins and hormones [3]. It has been reported that lipids have the function of sparing protein in feed, reducing nitrogen excretion, and improving the environment in aquaculture [4]. Lipids can also be further digested and absorbed in the presence of bile acids (BAs). Therefore, identifying suitable feed additives, such as BAs, that can promote growth, enhance immunity, improve antioxidant capacity, and protect liver and intestine is significant in aquaculture.

Recent studies on BAs have significantly advanced our understanding of their significant positive effects on growth performance and health in aquatic animals, such as large yellow croaker (*Larimichthys crocea*) [4], leopard coral grouper (*Plectropomus leopardus*) [5, 6], grass carp (*Ctenopharyngodon idellus*) [7], Largemouse bass (*Micropterus salmoides*) [8, 9], white shrimp (*Litopenaeus vannamei*) [10–12], among others. Thus, BAs are regarded as a promising growth-promoting and lipid-lowering feed supplement for the sustainable aquaculture industry [13].

Synthesized in the liver of higher animals, BAs have been widely applied as simple lipid emulsifiers and complex metabolic regulators, playing prominent roles in the metabolic systems of farmed shrimps [2, 10–12, 14, 15]. For instance, Su et al. [10] demonstrated that dietary supplementation of 0.2–0.3 g kg^−1^ BAs could improve lipid digestion and absorption, leading to enhanced growth performance in *L. vannamei*. Similarly, Li et al. [12] reported that BAs improved the intestinal microbiota and enhanced the growth performance in shrimp. BAs act as binding agents for sodium and potassium salts, forming bile salts that then combine with phospholipids and cholesterol to create mixed micelles. This process enhances the solubility of cholesterol micelles while facilitating the digestion and absorption of lipids and fat-soluble vitamins [16]. BAs could also promote lipid metabolism by regulating lipid emulsification, lipase activity, and the digestion and absorption of lipid substances [17]. Meanwhile, bidirectional regulation enables BAs to maintain the body's cholesterol balance [18]. In addition, BAs have the ability to effectively decrease harmful bacteria populations, mitigate gastrointestinal disorders, and minimize intestinal endotoxins [19].

However, shrimp lack the ability to synthesize BAs de novo and must obtain them from dietary sources [12]. Despite this limitation, a variety of methods, including chemical and biological approaches, can be used to obtain a mixture of BAs, such as hyodeoxycholic acid, chenodeoxycholic acid and cholic acid. It is important to note that evaluating the function of BAs derived from different components may not be appropriate. Therefore, establishing standardized production, extraction, and detection methods for different components and evaluating their functions in shrimp is crucial. In our previous work, we successfully produced a type of fermented bile acids (FBAs) through a biological method, demonstrating improvements in growth performance and intestinal health by altering metabolic profiles and intestinal microbiome in *M. salmoides* [20]. However, the potential role of FBAs in shrimp remains unexplored.

Based on the above background, two types of BAs named FBAs and 170HDa were first produced by fermenting the pig bile with an engineered *Escherichia. coli* (*E. coli*), then the effects of dietary FBAs and 170HDa on *L. vannamei* were investigated from the aspects of growth performance, lipid metabolism, pathology of hepatopancreas and intestine, nonspecific immunity, and intestinal microbiota.

## 2. Materials and Methods

### 2.1. FBAs and 170HDa

The genetically engineered *E. coli* containing bile salt hydrolase was constructed by Chem-Bright Bioengineering Co., Ltd, which could catalyze bile salt into BAs mainly composed of hyodeoxycholic acid [20]. The 170HDa was first obtained after a series of processes, including *E. coli* fermentation, sterilization, decolorization, magnesium salt formation, magnesium removal, and drying. Following this, FBAs were obtained from the residual of 170HDa. The compositions of FBAs and 170HDa were determined by high-performance liquid chromatography. All these processes were performed by the Chinese patent (No. CN202311158729 and No. 202311158709).

### 2.2. Shrimp and Experimental Conditions

Experimental *L. vannamei* (*L. vannamei*) were obtained from Leyuhui Ecological Agriculture Development Co., Ltd (Jining, China) and acclimated for 14 days. Subsequently, shrimp was cultured, as previously described [21]. In brief, shrimps with an average weight of 3.46 ± 0.24 g were randomly distributed into individual cages (100 × 100 × 140 cm, 100 shrimp in each cage and three cages per group). The shrimp were fed three times daily (6 : 00, 12 : 00, and 18 : 00) at a rate of 5%–7% of the biomass until obvious satiation was observed. During the experimental period, one-third of water in each cage was changed every day, using natural water sourced from Dawen River, which was monitored daily for quality parameters, including temperature (27.0 ± 2°C), dissolved oxygen (6.0 ± 1 mg L^−1^), pH (8.0 ± 0.5), and salinity (0.7 ± 0.1‰). The concentrations of total ammonia nitrogen (<0.4 mg L^−1^) and nitrite (<0.1 mg L^−1^) were determined three times per week using standardized methods.

### 2.3. Experimental Diets

The composition of the basal diet is shown in Table 1. Five experimental diets were prepared with FBAs levels at 0.02% (A1), 0.03% (A2), 0.04% (A3), 0.05% (A4), and 0.06% (A5), and five diets were prepared with 170HDa levels at 0.02% (B1), 0.03% (B2), 0.04% (B3), 0.05% (B4), and 0.06% (B5). A positive diet (P) was prepared using commercial BAs at a level of 0.04%, while a negative diet (N) contained no added BAs. The concentrations of FBAs and 170HDa (0.02%−0.06%) were chosen based on preliminary experiments and previous studies, which demonstrated optimal growth and health benefits within this range for aquatic species [10, 14, 15, 20].

All diets were prepared as pellets (1.0 mm diameter) using a laboratory feed-pelletizer and deposited at 20°C until usage [21].

### 2.4. Sample Collection

Before the start of the experiment, 15 *L. vannamei* were randomly selected from each group, and hemolymph, hepatopancreas, midgut, and intestinal contents were collected as the pretreatment group. After 30 or 60 days, 15 shrimps were sampled randomly from each cage, and a mixed sample with five shrimp was used. A sterile syringe containing 250 μL of 0.5M EDTA was used to withdraw about 250 μL of hemolymph from the ventral sinus of shrimp. After centrifugation, the supernatant was utilized for assessing parameters associated with immune response and hemolymph biochemistry. The entire intestine and hepatopancreas of each shrimp were collected and stored at −80°C. For analysis of intestinal microbiota, the intestines of 10 shrimps were employed. Hepatopancreas were kept for enzyme activity assays.

### 2.5. Growth Performance Analysis

The growth parameters were calculated using the following formulas:

Weight gain (WG, %) = (FW—IW)/IW × 100;

Specific growth rate (SGR, % day^−1^) = [ln (FW)−ln (IW)]/t × 100;

IW (g) represents the initial weight and FW (g) represents the final weight (FW).

### 2.6. Histopathological Examination

Following established methods, we aimed to evaluate the impacts of FBAs and 170HDa on the health status of the intestinal and hepatopancreas tissues by analyzing various indicators [10]. On the 60th day of the experiment, five *L. vannamei* were randomly selected from each group, and the midgut and intact hepatopancreas were collected postdissection. Briefly, the tissues were fixed in 4% formaldehyde, followed by ethanol gradient dehydration, clearing in toluene, and transparency treatment in xylene before embedding in paraffin. The sections were cut to a thickness of 5 μm. Hematoxylin-eosin (H&E) staining was applied, and image data were gathered under a microscope(Nikon, ECLIPSE, Japan)for further analysis.

### 2.7. Biochemical Indicators and Enzyme Activity

The levels of alanine aminotransferase (ALT), aspartate aminotransferase (AST), high-density lipoprotein (HDL-C), low-density lipoprotein (LDL-C), triglycerides (TG), *α*-amylase (*α*-AMS), alkaline phosphatase (AKP), and acid phosphatase (ACP) in the hemolymph were determined following the instructions provided with the relevant kits from Nanjing Jiancheng Bioengineering Institute, China.

### 2.8. Quantitative Real-Time PCR Analysis

RNA extraction from the intestine and hepatopancreas was conducted using the Steady Pure Universal RNA Extraction Kit II (Takara, Japan). After assessing the purity of the RNA with agarose gel electrophoresis, the concentration was detected by spectrophotometry. A PrimeScriptTM RT Reagent Kit with gDNA Eraser was used to transcribe RNA (400 ng/μL) into cDNA. Real-time quantitative PCR was conducted by combining 5 μL of SYBR Green Master Mix (Takara), 0.5 μL of upstream and downstream primers (Table 2), 1 μL of sample cDNA (150 ng/μL), and 3 μL of water, resulting in a total reaction volume of 10 μl, on a LightCycler 480 instrument (Roche Applied Science) [19]. The qPCR reactions were conducted using the following thermal cycling conditions: one cycle of 95°C for 10 min, 40 cycles of 95°C for 15 s and 60°C for 30 s, and 72°C for 30 s. *β*-actin served as an internal control for calculating the expression levels of the target genes using the 2^-*ΔΔ*CT^ method [22].

### 2.9. Intestinal Microbiota Analysis

The analysis of intestinal microbiota involved identifying the V3-V4 region of the 16S rRNA gene using high-throughput sequencing. Total DNA of intestinal bacterial community was extracted using a soil DNA extraction kit (TIANGEN, China) and quantified using a NanoDrop ND-2000 spectrophotometer (Thermo Science, USA). PCR amplification was performed using primers targeting the 16S V3-V4 region (341F and 806R), with the sequence being 341 F: 5′-CCTACGGGNGGCWGCAG-3′, and 806R: 3′-GGACTACHVGGGTATCTAAT-5′. The PCR reaction system was 25 µL, followed by high-throughput sequencing using Illumina-NovaSeq-6000. The amplicons were separated by 2% agarose gel electrophoresis, purified with the AxyPrep DNA Gel Extraction Kit (Axygen Biosciences, USA), pooled equimolarly, and subjected to paired-end sequencing (2 × 300) on an Illumina MiSeq platform using MiSeq Reagent Kit v3 at Shanghai Personal Biotechnology Co., Ltd (Shanghai, China).

Microbiome bioinformatics was mainly performed with QIIME 2 2019.4, with OTU clustering conducted following the Vsearch (v2.13.4) pipeline [23, 24]. Briefly, raw sequence data were demultiplexed using the demux plugin, followed by primers cutting with cutadapt plugin. Sequences were merged, filtered, and dereplicated using fastq_mergepairs, fastq_filter, and derep_fulllength functions in Vsearch. All unique sequences were clustered at 98% (via cluster_size), followed by chimera removal (via uchime_denovo). At last, nonchimera sequences were reclustered at 97% to generate OTU representive sequences and an OTU table. Representive sequences were aligned with mafft and used to construct a phylogeny with fasttree2. Alpha-diversity metrics (Chao1, Shannon, Simpson), Pielou's evenness and Good's coverage, along with beta diversity metrics (weighted UniFrac, unweighted UniFrac, Jaccard distance, and Bray-Curtis dissimilarity), were estimated using the diversity plugin, with samples rarefied to 1000 sequences per sample. Taxonomy was assigned to amplicon sequence variants (ASVs) using the classify-sklearn naïve Bayes taxonomy classifier in feature-classifier plugin against the Silva v132 99% OTUs reference sequences [24, 25].

### 2.10. Statistical Analysis

Statistical analysis was performed using SPSS 27.0 (USA). One-way analysis of variance (ANOVA) was conducted to assess the effect of different dietary BA levels on the measured parameters, followed by Tukey's HSD test. Linear and/or quadratic trends were measured by contrasts of orthogonal polynomials. In addition, an independent-sample *t*-test was conducted for the results of the alpha diversity-related indices. Statistical significance was established at *p* < 0.05 or *p* < 0.01.

## 3. Results

### 3.1. Composition and Content of FBAs and 170HDa

**Figure 1:**
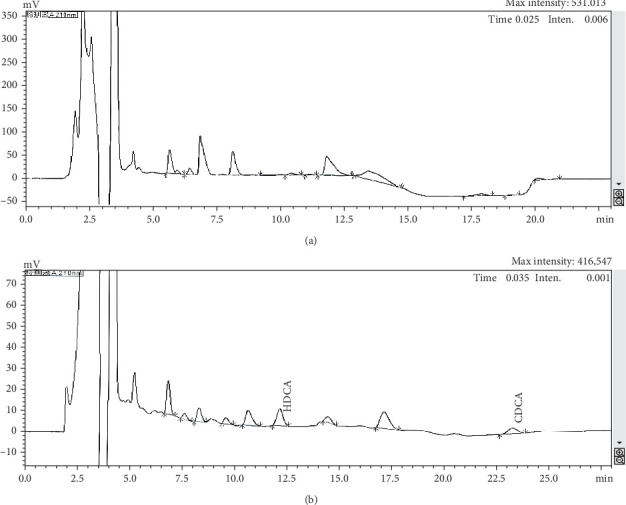
Comparison of bile was before and after fermented by *E. coli* and determined by high-performance liquid chromatography. (A) Raw bile, (B) Hydrolyzed bile for 4 h. CDCA, chenodeoxycholic acid; HDCA, hyodeoxycholic acid.

No free hyodeoxycholic or free chenodeoxycholic was detected in the bile (Figure 1A). However, after 4 h of fermentation using *E. coli*, free hyodeoxycholic and free chenodeoxycholic were present (Figure 1B). The process of bile was catalyzed from conjugated BAs into free BAs over time. The composition of FBAs and 170HDa is different. In FBAs, the total content of cholic acid was 38.90%, which included 20.80% chenodeoxycholic acid, 10.50% hyodeoxycholic acid, 3.96% 3,7,12-trihydroxycholic acid, and 3.64% 3,6,7-trihydroxycholic acid. In contrast, the total content cholic acid in 170HDa was 84.64%, comprising 69.88% hyodeoxycholic acid, 3.14% chenodeoxycholic acid, 9.58% 3,7,12-trihydroxycholic acid, and 2.04% 3,6,7-trihydroxycholic acid.

### 3.2. Growth Performance of Shrimp

As shown in Table 3, different levels of dietary supplementation with FBAs and 170HDa significantly affected the growth performance of shrimp (*p* < 0.05). After 30 days of feeding, the FW, WG, and SGR in treatment groups (except A1) were significantly increased (*p* < 0.05) compared to the negative control group, but these data were lower when compared to the positive group with commercial BAs. After 60 days of feeding, the FW, WG, and SGR were significantly higher in groups A3 and B2–B4 than the negative control group and significantly higher in group B3 than the positive control group.

### 3.3. Intestine Histology Observation

**Figure 2:**
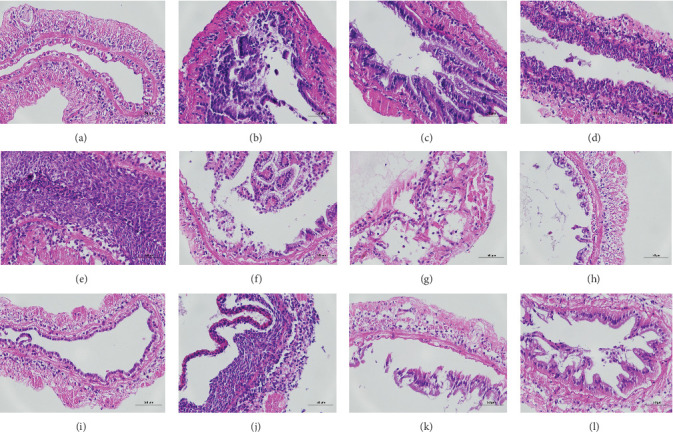
Histological sections of the mid-intestine of white shrimp (*L. vannamei*) from different treatment groups. (A) A1, (B) A2, (C) A3, (D) A4, (E) A5, (F) B1, (G) B2, (H) B3, (I) B4, (J) B5, (K) P, (L) N.

The histopathological examination of the shrimp intestine is illustrated in Figure 2. In group A1, the intestinal structure was intact without apparent lesions (Figure 2A). Similarly, group A2 exhibited no significant pathological changes (Figure 2B). A3 showed mild infiltration of inflammatory cells (Figure 2C), while group A4 displayed slight thinning of the intestinal wall without apparent lesions (Figure 2D). Mild disorganization of muscle fibers was observed in group A5 (Figure 2E). In group B1, the midgut microstructure appeared intact and healthy (Figure 2F), and group B2 displayed slight structural damage, although no significant lesions were noted (Figure 2G). Group B3 maintained an intact epithelial structure with a mild increase in lymphocytes (Figure 2H). Group B4 tended toward an intact structure without apparent lesions (Figure 2I), while group B5 exhibited thickening of the intestinal wall, with the upper layer of the intestinal mucosa remaining intact (Figure 2J). The commercially treated BA group shows no significant pathological changes, trending toward a healthy state (Figure 2K). In contrast, the group without BA supplementation showed a clear inflammatory response, characterized by thinning of the intestinal wall and decreased intestinal barrier function (Figure 2L). Compared to the control group, the FBAs-treated group maintained intestinal health, while the 170HDa-treated group demonstrated an increase in intestinal tract thickness, further promoting intestinal health. Notably, the 170HDa-treated group exhibited a more pronounced beneficial effect on intestinal health compared to the FBAs.

### 3.4. Hepatopancreas Histology Observation

**Figure 3:**
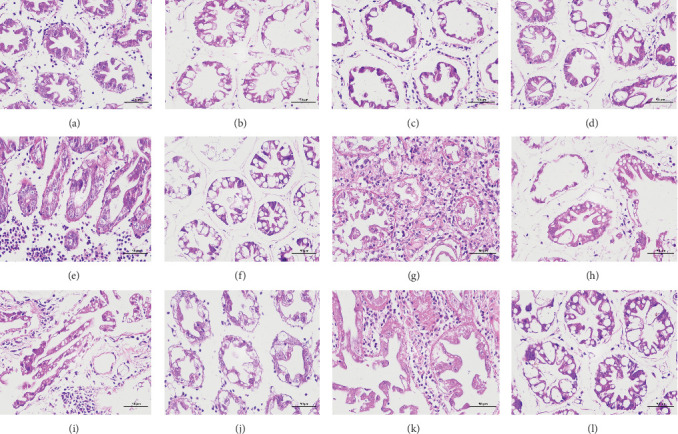
Histological sections of the hepatopancreas tissues of white shrimp (*L. vannamei*) from different treatment groups. (A) A1, (B) A2, (C) A3, (D) A4, (E) A5, (F) B1, (G) B2, (H) B3, (I) B4, (J) B5, (K) P, (L) N.

The pathological examination of the hepatopancreas are presented in Figure 3. In group A1, the hepatopancreatic tissue comprised well-developed and multibranched tubules without apparent lesions (Figure 3A). Similarly, group A2 showed no significant pathological changes, aside from occasional cytoplasmic vacuolization in the hepatopancreatic tubular epithelium (Figure 3B). Group A3 exhibited mild inflammatory cell infiltration (Figure 3C), while in group A4, slight vacuolization occurs without apparent lesions (Figure 3D). In group A5, the local arrangement of hepatopancreatic tubules becomes irregular, with a small amount of tubular necrosis observed (Figure 3E). In group B1, lumens appeared pentagonal or star-shaped, and the connective tissue was rich in blood sinuses, showing no obvious abnormalities (Figure 3F). Similarly, group B2 displayed a disrupted local arrangement of hepatopancreatic tubules, with increased tubular necrosis, nuclear fragmentation, and enhanced cytoplasmic acidophilia (Figure 3G). Group B3 displayed an irregular arrangement of hepatopancreatic tubules (Figure 3H). In group B4, there was a small amount of shedding of the epithelium of the hepatopancreatic tubules and nuclear fragmentation (Figure 3I). Group B5 exhibited more inflammatory cell infiltration in the interstitium (Figure 3J). In the market BA treatment group, a small amount of inflammatory cell infiltration was observed in the interstitium (Figure 3K). In the group without added BA, the local arrangement of hepatopancreatic tubules was disrupted, with more shedding of the epithelium, nuclear fragmentation, enhanced cytoplasmic acidophilia, and a higher amount of inflammatory cell infiltration (Figure 3L).

Compared to the control group, the FBAs treatment group showed a milder inflammatory response. In contrast, the 170HDa treatment group increased the frequency of inflammatory responses compared to the control group. Moreover, the 170HDa treatment group demonstrated a more pronounced advantage in promoting inflammatory reactions compared to FBAs.

### 3.5. Biochemical Measurements of Hemolymph

Table 4 shows the effect of dietary FBAs and 170HDa at different levels on metabolism-related biochemical parameters in hemolymph of shrimp for 30 or 60 days. No significant difference was found in HDL, LDL, and AMS only for 60 days between different treatments.

After 30 days of feeding, the activities of AST in A1–A4, B1–B3 groups were significantly lower (*p* < 0.05) compared to both the positive and negative control groups. For ALT, BAs and 170HDa significantly reduced the enzyme activities in most treatment groups compared to the negative control, with A4 and B3 showing significant reductions (*p* < 0.05) relative to the positive control. The levels of AMS were enhanced by dietary supplementation of FBAs and 170HDa (except in A1). TG contents in hemolymph of shrimp were decreased by different treatments, with only B3 showing significantly lower TG levels compared to the positive control.

After 60 days of feeding, the treatment groups significantly decreased the activities of AST and ALT compared to control group. The effects on TG levels varied, with higher levels in A1–A3, B3 and lower levels in B1, B2, and B5.

### 3.6. Effects on Nonspecific Immune Responses

The results of nonspecific immune are shown in Tables 4–6. The highest AKP activity (AKPa) of hemolymph appeared in group A4 after 30 days of FBAs treatment and in group A2 after 60 days (Tables 4 and 5). Generally, AKPa of hemolymph in FBAs-treated shrimp was higher than 170HDa-treated groups and control groups (*p* < 0.05) (Tables 4 and 5). The highest ACP activity (ACPa) of hemolymph appeared in A5 group after 30 days and in A3 group after 60 days of FBAs treatment (Tables 4 and 5). Overall, ACPa of hemolymph in FBAs-treated shrimp was comparable to that in 170HDa-treated and control groups (Tables 4 and 5).

Gene expression levels of AKP in the hepatopancreas were similar with those observed in hemolymph (Tables 4–6), with the highest expression level in group A4 after 60 days of FBAs treatment. The highest expression level of antilipopolysaccharride factor (ALF) in the hepatopancreas appeared in group B5 after 60 days of FBAs treatment. However, the expression levels of ALF in other FBAs and 170HDa-treated groups were significantly lower than those in the positive control group (*p* < 0.05) and notably lower than in the negative control group (*p* < 0.05). The highest gene expression level of prophenoloxidase (proPO) in the hepatopancreas appeared in the negative group after 60 days. Furthermore, the gene expression levels of proPO in the hepatopancreas were significantly lower in FBAs-, 170HDa-, and BAs-treated groups compared to the negative control group (*p* < 0.05). The highest expression level of alpha-2-macroglobulin (*α*2M) in the hepatopancreas appeared in the negative group after 60 days, with expression levels in FBAs-, 170HDa-, and BAs-treated groups significantly lower compared to the negative control (*p* < 0.05). Meanwhile, the gene expression levels of *α*2M in the hepatopancreas of 170HDa-treated groups were significantly higher than those in FBAs-treated group (*p* < 0.05).

### 3.7. Intestinal Microbiota Analyses

**Figure 4:**
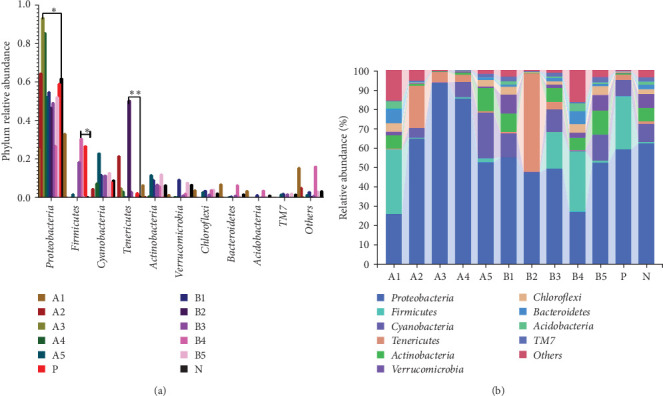
Relative abundance of intestinal bacteria of *L. vannamei* at the phylum level. Upright bars denote the mean ± SEM. (A) This bar chart shows the relative abundance of taxa at the phylum level across different groups. Statistical annotations indicate significant differences between groups. (B) This stacked bar chart illustrates the composition of taxa at the phylum level for each group, with different colors representing various phyla. *⁣*^*∗*^Indicates a significant (*p* < 0.05). *⁣*^*∗∗*^Indicates highly significant (*p* < 0.01).

The abundance of microbiota at the phylum level is depicted in Figure 4a, b, with Proteobacteria, Firmicutes, Cyanobacteria, and Tenericutes being the main phyla. Compared to the negative control, a significant increase in groups A3–A4 (*p* < 0.05) and a significant decrease in groups A1 and B4 (*p* < 0.05) were observed in the abundance of Proteobacteria. Intragroup comparisons revealed a significant decrease in group A3 (*p* < 0.05) and a highly significant decrease in group B4 (*p* < 0.01) compared to the other experimental groups. Significant increase in the abundance of Firmicutes was found in groups B3 and B4 (*p* < 0.05) compared to the negative control.

**Figure 5:**
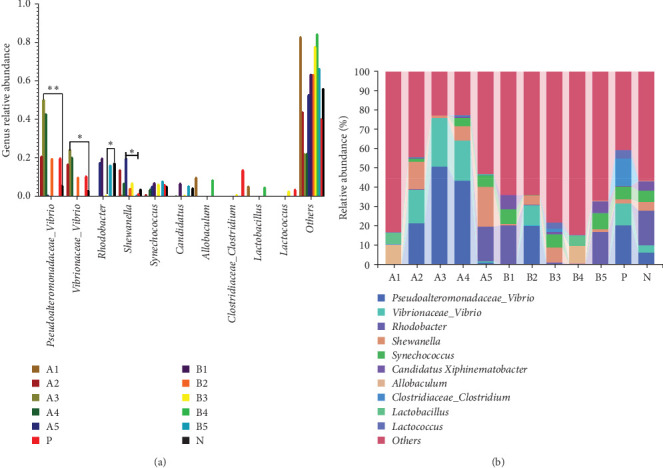
Relative abundance of intestinal bacteria of *L. vannamei* at the genus level. Upright bars denote the mean ± SEM. (A) This bar chart shows the relative abundance of taxa at the genus level across different groups. Statistical annotations indicate significant differences between groups. (B) This stacked bar chart illustrates the composition of taxa at the genus level for each group, with different colors representing bacterial genera. *⁣*^*∗*^Indicates a significant (*p*  < 0.05), *⁣*^*∗∗*^Indicates highly significant (*p*  < 0.01).

Tenericutes were significantly increased in the B2 group compared to the negative control and other experimental groups (*p* < 0.01). Cyanobacteria in group A5 was significantly elevated compared to the negative group and other experimental groups (*p* < 0.05). At the genus level, the main intestinal bacteria were *vibrio*, *Rhodobacter*, and *Shewanella* (Figure 5). The abundance of *V. vibrio* was significantly higher in group A3 than that in the control group and groups B1, B4, and B5 (*p* < 0.01). Within-group comparisons showed a significant decrease in A5, B1, B4, and B5 (*p* < 0.05). The relative abundance of *Rhodobacter* significantly increased in B3 group compared to the control group, but no significant changes were observed compared to other experimental groups. *Shewanella* significantly increased in group A5 than the other groups (*p* < 0.05).

**Figure 6:**
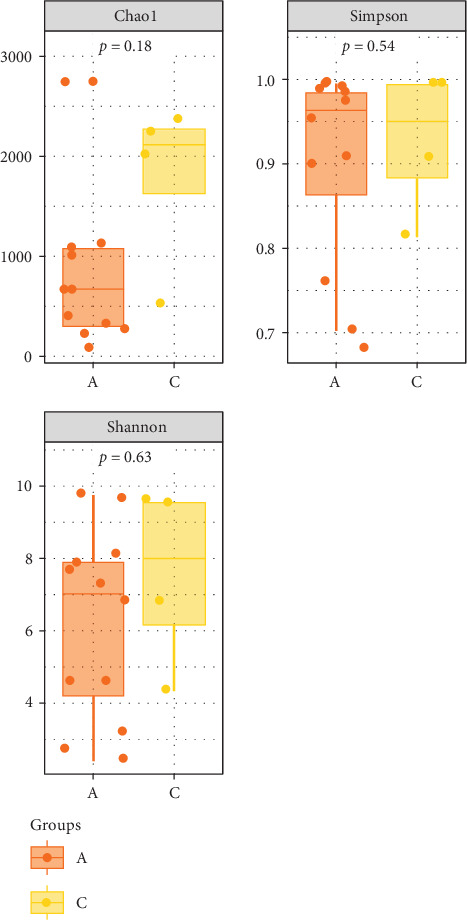
Alpha species diversity analysis. A: FBAs and 170HDa groups, C: Pre-sampling group before experimentation. The *p*-value represents the abundance of species in the community. FBAs, fermented bile acids.

**Figure 7:**
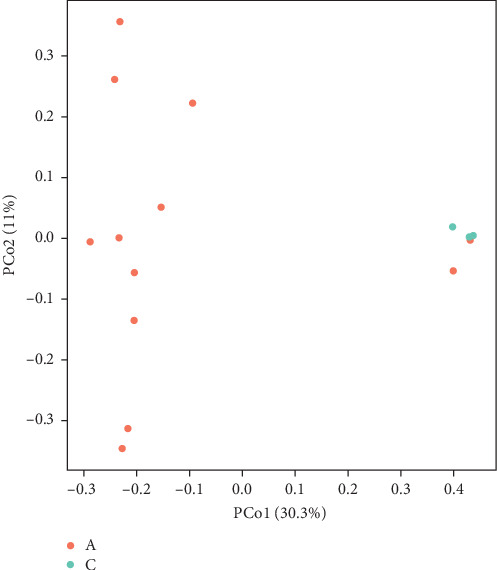
Principal coordinate analysis (PCoA) of intestinal microbial community of *L. vannamei*.

The Chao 1, Simpson, and Shannon are shown in Figure 6. Adding FBAs and 170HDa enhanced the diversity of the intestinal microbiota without significant difference compared to the control groups (*p* > 0.05). In groups A1–B5, species diversity in the intestinal of shrimp increased as the FBAs and 170HDa concentration increased, while it decreased in the control group. Additionally, based on weighted UniFrac distance, principal coordinate analysis (PCoA in Figure 7) showed a distance separation between bacterial communities in groups BAs and 170HDa when compared to negative control group. These findings suggested a significant difference in community structure with regard to beta diversity after dietary supplementation.

**Figure 8:**
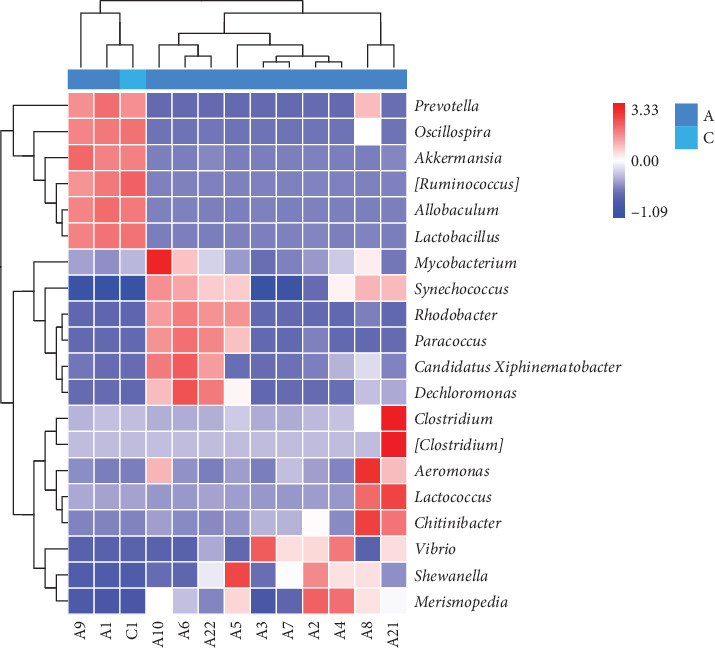
Changes in the intestinal microbiota at the genus level in the C1, A1-B5 and A21-A22 groups. (The heatmaps of the specimens show the relative abundances of the main identified bacteria at the genus taxonomic level. Red indicates a higher relative abundance, whereas blue indicates a lower relative abundance.) A6: B1, A7: B2, A8: B3, A9: B4, A10: B5, A21: P, A22: N, C1: Presampling group before experimentation.

In order to further investigate the differences in bacterial communities, a bacterial heatmap analysis of the top 20 most abundant genera in the intestine is shown in Figure 8. Significant increases in *Prevotella*, *Oscillospira*, *Akkermansia*, *Allobaculum*, *Clostridium*, *Aeromonas*, *Lactococcus*, and *Chitinibacter* were observed after treatment with BAs (*p* < 0.05), while the proportion of *Synechococcus* and *Rhodobacter* decreased significantly (*p* < 0.05).

**Figure 9:**
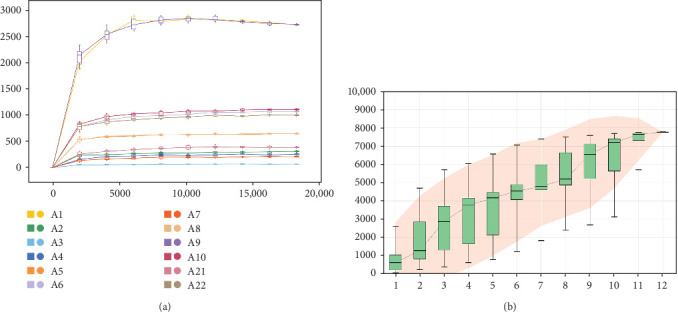
(A) Rarefaction curve analysis of intestinal samples in *L. vannamei* from different treatment groups. (B) The species accumulation boxplot. The horizontal axis represents the number of samples, and the vertical axis represents the number of species found. A6: B1, A7: B2, A8: B3, A9: B4, A10: B5, A21: P, A22: N.

**Figure 10:**
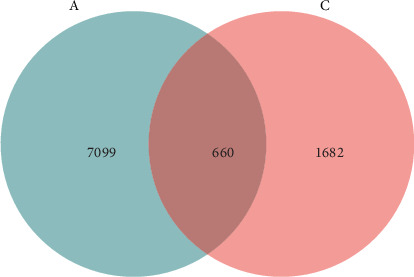
Venn diagram of OTUs comparison. A: FBAs and 170HDa groups, C: Pre-sampling group before experimentation. FBAs, fermented bile acids.

The analysis of rarefaction curve is presented in Figure 9A. As sequencing depth increases, the curve begins to plateau, indicating that the sequencing results sufficiently capture the structure and composition of the shrimp gut microbial community. Figure 9B displayed the box plot analysis, where the rising position of the box plots within a certain range suggests a high species richness in the community. Once the box plots level off, the number of species no longer increases significantly with sample size, demonstrating that the sampling effort is adequate and the data can be reliably analyzed. The Venn diagram analysis (Figure 10) revealed a total of 660 OTUs identified across both treatments. The FBAs and 170HDa groups uniquely contained 7099 OTUs, while the presampling group before the experiment had 1682 OTUs. This revised order first presents the rarefaction curve and box plot analyses to assess sampling adequacy, followed by the Venn diagram and OTU counts, making the results clearer and more logically structured.

## 4. Discussion

### 4.1. The Advantage of FBAs and 170HDa

BAs are a type of feed additive commonly extracted from bile using chemical method in traditional saponification processes. Previous studies have often overlooked the preparation methods for dietary BAs, suggesting that many of these compounds may have been produced using conventional techniques [2, 10–15]. In our research, we introduced an innovative approach to obtain FBAs and 170HDa by hydrolyzing conjugated BAs in bile into free BAs utilizing genetically engineered *E. coli*, as previously described [20]. The resulting free BAs consisted of hyodeoxycholic acid, chenodeoxycholic acid, 3,7,12-trihydroxycholic acid, and 3,6,7-trihydroxycholic acid. This biological method has been shown to reduce consumption and save energy and time compared to traditional chemical processes, by eliminating the need for excessive alkali and reducing process time from 24 to 4 h [20].

### 4.2. Effect of FBAs and 170HDa on the Growth Performance of Shrimp

BAs have been well-documented to enhance the growth performance of white shrimp when applied at appropriate levels [10, 14, 15, 21]. Our study confirmed this promoting effect, as dietary supplementation with FBAs and 170HDa improved the growth performance, including FW, WG, and SGR, with an optimal dietary level at 0.04%. Previous findings also indicated that dietary supplementation with 0.15 or 0.2–0.3 g kg^−1^ of BAs promotes the growth performance of shrimp [10, 26]. Contrary to these studies, supplementation with 0.08% chenodeoxycholic acid in a low fishmeal diet did not affect shrimp growth [11]. The growth-promoting effect of BAs is highly related to factors such as feed species, basal diet conditions, and BAs type and levels [27].

FBAs and 170HDa are two distinct types of BAs, and the varying results obtained in our study indicate that they have different growth-promoting effects. FBAs can improve the shrimp growth in a short term, but the effectiveness may diminish over longer durations, such as 2 months. Conversely, the growth-promoting effect of 170HDa is obviously better than that of FBA, and even superior to commercial BAs at the same concentration, indicating its potential as a new functional additive in shrimp aquaculture.

### 4.3. Effect of FBAs and 170HDa on the Intestinal and Hepatopancreas Damage of Shrimp

BAs are currently employed extensively as novel feed additives in aquaculture [28, 29]. Classified as amphoteric sterol compounds, BAs contain both lipophilic alkyl groups and hydrophilic hydroxy and carboxy groups, thus exhibiting dual properties that facilitate fat emulsification. This emulsification process enhances the interaction between fats and lipases, thereby improving the digestion and absorption of lipid substances. Crustaceans lack the ability to synthesize endogenous BAs, necessitating the intake of BAs during various growth stages to fulfill nutritional requirements. For crustaceans, BAs serve as essential steroidal substances and vital nutritional components for normal growth. In our research, we prepared two types of BAs, named FBAs and 170HDa, through the fermentation of pig bile with engineered *E. coli*. Subsequently, these BAs were employed to enhance the intestinal health of white shrimp.

BAs play a critical role in regulating cholesterol metabolism and maintaining stable cholesterol levels [30]. Cholesterol serves as a crucial precursor for various physiologically active substances in the body, including ecdysteroids, sex hormones, and vitamin D [31, 32], all of which significantly influence growth, molting, and carbohydrate and lipid metabolism in the exoskeletons of crustaceans. Consequently, BAs are crucial for maintaining intestinal health by promoting cell growth and preventing cell death in the intestinal epithelium [33]. Histopathological examination of the intestine indicated that the shrimp-fed diets containing FBAs had no significant pathological changes, aside from slight infiltration of inflammatory cells, possibly due to sampling influences. Nevertheless, this group demonstrated significantly improved intestinal health compared to the FBAs alone. In contrast, the control group, without BAs in their diets, experienced severe pathological changes. This observation underscores the substantial enhancement of intestinal health in crustaceans, receiving BAs prepared through varied processes, highlighting the pivotal role of BAs in preserving gastrointestinal health in aquatic animals. Pathological sections of the hepatopancreas revealed that the group fed with FBAs did not exhibit significant inflammatory responses, and all experimental groups appeared relatively healthy. Severe pathological changes in individual experimental groups may also be attributed to sampling factors. However, the group receiving 170HDa-containing feed showed a significant increase in inflammatory responses in the hepatopancreas compared to those receiving FBAs. The control group not receive BA-containing feed exhibited severe pathological changes, indicating that BAs prepared through different processes significantly enhance the immune response in crustaceans. This highlights the importance of 170HDa in promoting inflammatory reactions for maintaining crustacean health.

### 4.4. Effect of FBAs and 170HDa on Shrimp Metabolism

AST and ALT levels in hemolymph are important biomarkers for evaluating liver health status [34]. In this study, no significant increase in AST and ALT activities was found, indicating that dietary supplementation at a level of 0.06% did not lead to liver injury. After 60 days of dietary supplementation with FBAs and 170HDa, there were no effects on AMS activity in white shrimp, consistent with findings where the addition of 0.2–0.5 g/kg BAs also showed similar results [10]. This lack of effect may be attributed to the shrimp's low digestion and utilization rates of carbohydrates [26]. TG is an important component in blood lipids and can reflect the body's lipid metabolism status. The fluctuated data in this study may indicate activation of lipid metabolism. BAs were suggested to regulate the lipid metabolism by promoting the digestion and absorption of dietary lipids after emulsifying fat and improving the activity of lipase [17].

### 4.5. Effect of BAs on the Immunity of Shrimp

Host immune responses, including components of the nonspecific immune system such as cytokines (e.g., AKP, ACP, ALF, proPO, and *α*2M), serve as primary defenses against pathogens [22, 35–37]. These immune mechanisms evolved from early intracellular molecules before the appearance of receptors and signaling cascades and play a key role in host defense [22, 36]. ACPa and AKPa in the nonspecific immune system represent macrophage activation and their intracellular digestion abilities for phagocytized antigens in invertebrate immunity [38, 39]. In this study, AKP activity in the hemolymph of shrimp treated with FBAs was generally higher than that in both the 170HDa-treated and control groups. However, ACP activity in the hemolymph of FBAs-treated shrimp was similar to that in the 170HDa and control groups. Meanwhile, gene expression levels of ALF and proPO in FBAs and 170HDa-treated groups were significantly lower than those in the positive control group, while being significantly lower than the negative control group. Gene expression levels of *α*2M in the hepatopancreas in 170HDa-treated groups were significantly higher than those in FBAs-treated group. In shrimp, ALF, proPO, and *α*2M contribute to building barriers that protect the host from pathogenic microorganisms, resist allergic reactions, and modulate immune responses [3, 32, 35]. In addition, BAs could improve liver function, antioxidant responses, and immunity in various cultured fish [4, 22].

### 4.6. Effect of FBAs and 170HDa on the Intestinal Microbiota of Shrimp

The dominant bacteria phyla detected in all treatment groups were Proteobacteria, Firmicutes, Cyanobacteria, and Tenericutes. Proteobacteria is the largest bacterial phylum, including numerous pathogenic species like *E. coli*, *Salmonella*, *Vibrio cholerae*, and *Helicobacter pylori*, as well as various free-living species capable of nitrogen fixation [40].

Firmicutes play a key role in metabolic activities in the intestinal tract, such as carbohydrate fermentation, nitrogenous substance utilization, and the biological transformation of BAs and sterols. The majority of intestinal bacteria undergo sugar fermentation, acquiring carbon and energy through carbohydrate molecule hydrolysis [41]. Experimental results showed a significantly higher abundance of Firmicutes in the 170HDa-treated group compared to the control group, further indicating the promoting effect of BAs from different sources on the intestinal health of white shrimp. Cyanobacteria constitute a group of sizable prokaryotic microorganisms with extensive evolutionary histories, characterized by their ability to perform oxygen-producing photosynthesis [42]. Tenericutes are notable for their probiotic qualities, exhibiting slow growth, a lack of a cell wall, inability to synthesize, and good biodegradability. In this study, Proteobacteria, Firmicutes, Cyanobacteria, and Tenericutes were found as the dominant phyla in the gut across all groups. Dietary supplementation with FBAs and 170HDa modified the intestinal microbiota structure of white shrimp, as evidenced by changes in *α*-diversity and PCA analysis. Proteobacteria significantly decreased, while Firmicutes, Tenericutes, and Cyanobacteria significantly increased compared to the control group. These findings indicate that FBAs and 170HDa can enhance the gut microbial community balance.

At the genus level, *Vibrio*, *Rhodobacter*, and *Shewanella* exhibited higher abundance in the groups treated with FBAs and 170HDa compared to the control group. *Vibrio*, commonly found in natural habitats such as soil and water, holds significant potential in medical applications due to their antioxidant properties, aiding in skin protection against free radical-induced damage and slowing the aging process. Additionally, *Vibrio* has regulatory roles in sebum secretion. The abundance of bacterial groups A3 and A4 in the FBAs treatment group was notably greater than that in the control group (*p* < 0.01). Conversely, the abundance of *Vibrio* in the 170HDa treatment group was lower than that in the control group, thus underscoring the potential superiority of 170HDa as a feed additive.

The genus *Rhodobacter* is characterized as Gram-negative with vesicular photosynthetic membranes. Its species can grow photoautotrophically by utilizing hydrogen sulfide as an electron acceptor and can also engage in photoheterotrophic growth under anaerobic conditions [43]. The abundances of groups B3 and B4 in the 170HDa treatment group were significantly lower than those in the control group, indicating a substantial impact of 170HDa on the gut microbiota of white shrimp. *Shewanella*, widely present in both freshwater and marine environments, is also commonly present in gut microbiota and can exhibit both planktonic growth and parasitic behavior on other organisms [44]. The abundance *Shewanella* in group A5 in the FBAs treatment group is significantly higher than that in the control group, suggesting that high-concentration fermented BAs may enhance the specificity of gut microbiota species.

## 5. Conclusion

*L. vannamei* is a typical kind of marine and freshwater shrimp known for its high muscle quality and rich protein content. The results of this study suggested that FBAs and 170HDa obtained through biological method were better than those derived from chemical method. Furthermore, dietary supplementation with FBAs and 170HDa improved growth performance and intestinal health by altering lipid metabolic profiles and intestinal microbes in white shrimp. This discovery holds significant potential for advancing the industry of white shrimp cultured with artificial diet.

## Figures and Tables

**Table 1:** Composition and concentration of nutrients in basal diet.

Ingredients	Contents
Imported fish meals	16.00
Domestic fish meals	16.00
Chicken meals	5.00
Shrimp shell powder	5.00
Peanut meals	10.00
43% soybean meal	20.00
Squid paste	2.50
Flour	19.35
Calcium monobasic phosphate	1.50
Phospholipid oil	3.00
Shrimp premix	1.00
Choline chloride	0.20
Antifungal agents	0.10
Sodium humate	0.30
Antioxidant	0.01
Aquatic complex enzymes	0.04
Total	100.00

**Nutrition facts**	**Numeric value**

Moisture content	9.10
CP	42.50
EE	8.00
Ash	12.40
Ca	1.80
P	1.60

**Table 2:** Primers for testing the immune-related genes in white shrimp by qPCR.

Primer name	Primer sequence (5′−3′)	Accession number	Amplicon size
AKP-F	CATCGTCAAGCAAGCCAAGAACATC	NC_000067.7	178
AKP-R	CTCGTGCTCGTCATTGCCTGTC		
proPO—F	CGGTGACAAAGTTCCTCTTC	NC_000913.3	183
proPO—R	GCAGGTCGCCGTAGTAAG		
A2M—F	CAATTACGCCTGGTACACGAGTCC	NC_000012.12	198
A2M—R	TCGCAAGGTCTGTTCTCAATGATGG		
ALF—F	ACTTCAATGGCAGGATGTGGTGTC	NC_054379.1	156
ALF—R	TCTAGCGTCGTCCTCCGTGATG		
*β*-actin-F	CCACGAGACCACCTACAAC	NW_023493663.1	162
*β*-actin-R	AGCGAGGGCAGTGATTTC		

**Table 3:** Effect of different dietary FBAs and 170HDa levels on growth performance in white shrimp.

Treatments	30 days			60 days		
	FW (G)	WG (%)	SGR (%)	FW (G)	WG (%)	SGR (%)
A1	4.14 ± 0.46^d^	19.68 ± 13.35^d^	0.58 ± 0.36^d^	5.87 ± 0.42^g^	69.77 ± 12.15^g^	0.88 ± 0.12^f^
A2	4.93 ± 0.57^c^	42.77 ± 16.47^c^	0.12 ± 0.36^c^	6.91 ± 0.53^ef^	99.92 ± 15.48^ef^	1.15 ± 0.13^de^
A3	5.81 ± 0.29^ab^	68.19 ± 8.51^ab^	1.73 ± 0.17^ab^	7.32 ± 0.33^cde^	111.97 ± 9.46^cde^	1.25 ± 0.07^cd^
A4	5.76 ± 0.43^ab^	66.66 ± 12.31^ab^	1.70 ± 0.25^ab^	7.18 ± 0.41^cdef^	107.72 ± 11.79^cdef^	1.22 ± 0.10^cde^
A5	5.62 ± 0.62^bc^	62.60 ± 17.82^bc^	1.60 ± 0.36^abc^	7.08 ± 0.58^def^	104.83 ± 16.77^def^	1.19 ± 0.14^cde^
B1	5.00 ± 0.55^c^	44.67 ± 16.04^c^	1.22 ± 0.36^c^	7.01 ± 0.49^ef^	102.77 ± 14.12^ef^	1.17 ± 0.12^de^
B2	5.54 ± 0.41^bc^	60.43 ± 11.76^bc^	1.57 ± 0.24^bc^	8.25 ± 0.42^ab^	138.92 ± 12.15^ab^	1.45 ± 0.09^ab^
B3	6.31 ± 0.50^ab^	82.72 ± 14.47^ab^	2.00 ± 0.26^ab^	8.74 ± 0.20^a^	153.05 ± 5.76^a^	1.55 ± 0.04^a^
B4	6.23 ± 0.67^ab^	80.27 ± 19.43^ab^	1.95 ± 0.37^ab^	7.83 ± 0.53^bcd^	126.75 ± 15.45^bcd^	1.36 ± 0.11^bc^
B5	6.14 ± 0.60^ab^	77.80 ± 17.50^ab^	1.91 ± 0.33^ab^	7.55 ± 0.25^bcde^	118.43 ± 7.18^bcde^	1.30 ± 0.05^bcd^
P	6.47 ± 0.87^a^	87.18 ± 25.16^a^	2.07 ± 0.45^a^	7.87 ± 0.74^bc^	127.88 ± 21.28^bc^	1.37 ± 0.15^bc^
N	3.99 ± 0.32^d^	15.57 ± 9.30^d^	0.47 ± 0.27^d^	6.53 ± 1.05^fg^	89.07 ± 30.29^fg^	1.04 ± 0.27^e^

*Note:* Data are showed with means ± SD for three replicates in each group, and the upper lowercase letters represent significantly different (*p* < 0.05).

Abbreviations: FBAs, fermented bile acids; FW, final weight; SGR, specific growth rate; WG, weight gain.

**Table 4:** Effect of different dietary FBAs and 170HDa levels on hemolymph biochemical parameters in white shrimp for 30 days.

Treatments	AST (U/L)	ALT (U/L)	HDL (mmoL/L)	LDL (mmoL/L)	AMS (U/dL)	TG (mmoL/L)	AKP(U/100 mL)	ACP(U/100 mL)
A1	6.42 ± 1.68^e^	51.56 ± 1.21^bc^	−0.17 ± 0.88	−0.16 ± 0.04	68.47 ± 0.97^c^	0.15 ± 0.03^bcd^	0.85 ± 0.12^b^	0.44 ± 0.06^e^
A2	6.33 ± 1.90^e^	51.11 ± 4.83^bc^	−0.62 ± 1.30	−0.13 ± 0.16	71.11 ± 0.84^abc^	0.15 ± 0.03^bcd^	0.65 ± 0.05^d^	0.51 ± 0.36^e^
A3	6.59 ± 0.84^e^	45.70 ± 5.01^d^	−1.30 ± 0.52	−0.23 ± 0.28	70.37 ± 0.48^abc^	0.16 ± 0.02^bc^	0.68 ± 0.09^cd^	1.32 ± 0.14^bc^
A4	2.99 ± 0.83^f^	29.59 ± 4.03^f^	−0.91 ± 1.46	−0.15 ± 0.06	70.26 ± 0.48^abc^	0.12 ± 0.01^cd^	1.29 ± 0.09^a^	1.57 ± 0.07^ab^
A5	17.00 ± 0.39^a^	58.58 ± 2.85^a^	−1.08 ± 0.94	−0.08 ± 0.00	71.64 ± 0.66^abc^	0.21 ± 0.04^a^	0.77 ± 0.07^bc^	1.75 ± 0.12^a^
B1	8.77 ± 1.20^d^	44.81 ± 2.96^de^	−1.13 ± 0.94	−0.13 ± 0.02	70.37 ± 2.95^abc^	0.11 ± 0.01^de^	0.70 ± 0.02^cd^	1.55 ± 0.13^ab^
B2	8.82 ± 1.13^d^	40.02 ± 2.48^e^	−0.96 ± 0.69	−0.15 ± 0.05	71.11 ± 0.84^abc^	0.19 ± 0.01^ab^	0.51 ± 0.06^e^	1.44 ± 0.21^ab^
B3	8.95 ± 1.38^d^	33.33 ± 0.97^f^	−0.45 ± 1.25	−0.21 ± 0.06	71.01 ± 1.02^abc^	0.08 ± 0.02^e^	0.14 ± 0.01^f^	0.85 ± 0.17^d^
B4	11.03 ± 0.23^cd^	48.52 ± 3.11^cd^	−1.19 ± 0.45	−0.11 ± 0.05	72.38 ± 0.32^ab^	0.17 ± 0.03^b^	0.24 ± 0.04^f^	1.61 ± 0.39^ab^
B5	14.62 ± 1.95^b^	43.87 ± 0.61^de^	−0.40 ± 1.63	−0.11 ± 0.06	72.59 ± 0.18^a^	0.12 ± 0.01^cd^	0.20 ± 0.03^f^	1.43 ± 0.06^ab^
P	11.67 ± 0.67^c^	44.53 ± 0.88^de^	−1.19 ± 0.74	−0.03 ± 0.02	53.12 ± 3.12^d^	0.15 ± 0.03^bcd^	0.25 ± 0.05^f^	1.07 ± 0.02^cd^
N	14.77 ± 1.26^b^	54.66 ± 1.06^ab^	−0.96 ± 0.59	−0.05 ± 0.06	68.89 ± 4.13^bc^	0.22 ± 0.02^a^	0.52 ± 0.05^e^	1.35 ± 0.16^bc^

*Note:* Data are showed with means ± SD for three replicates in each group, and the upper lowercase letters represent significantly different (*p* < 0.05).

Abbreviations: ACP, acid phosphatase; AKP, alkaline phosphatase; ALT, alanine aminotransferase; AMS, *α*-amylase; AST, aspartate aminotransferase; FBAs, fermented bile acids; HDL, high-density lipoprotein; LDL, low-density lipoprotein; TG, triglycerides.

**Table 5:** Effect of different dietary FBAs and 170HDa levels on hemolymph biochemical parameters in white shrimp for 60 days.

Treatments	AST (U/L)	ALT (U/L)	HDL (mmoL/L)	LDL (mmoL/L)	AMS (U/dL)	TG (mmoL/L)	AKP(U/100 mL)	ACP(U/100 mL)
A1	10.98 ± 0.49^bc^	30.96 ± 2.83^cd^	0.03 ± 0.07	−0.11 ± 0.05	68.85 ± 1.68	0.24 ± 0.01^b^	0.66 ± 0.01^b^	1.25 ± 0.19^de^
A2	11.02 ± 1.66^bc^	37.19 ± 1.57^b^	0.00 ± 0.09	−0.24 ± 0.14	66.75 ± 0.71	0.25 ± 0.03^b^	0.76 ± 0.02^a^	1.66 ± 0.13^b^
A3	10.63 ± 1.36^c^	39.84 ± 2.07^b^	0.03 ± 0.04	−0.13 ± 0.06	65.32 ± 3.19	0.31 ± 0.02^a^	0.63 ± 0.03^b^	2.59 ± 0.15^a^
A4	7.67 ± 1.32^e^	26.38 ± 2.42^ef^	−0.03 ± 0.00	−0.08 ± 0.07	68.01 ± 1.03	0.19 ± 0.01^de^	0.55 ± 0.03^c^	1.29 ± 0.13^de^
A5	8.41 ± 1.33^de^	27.78 ± 1.99^de^	0.03 ± 0.07	−0.08 ± 0.02	68.01 ± 1.36	0.22 ± 0.01^bc^	0.64 ± 0.04^b^	1.43 ± 0.09^cd^
B1	4.57 ± 1.23^f^	14.04 ± 0.63^h^	0.07 ± 0.10	−0.13 ± 0.06	69.36 ± 0.51	0.15 ± 0.01^gh^	0.56 ± 0.04^c^	0.95 ± 0.13^g^
B2	10.04 ± 0.34^cd^	32.72 ± 1.98^c^	−0.03 ± 0.02	−0.11 ± 0.03	66.11 ± 3.19	0.16 ± 0.01^fg^	0.32 ± 0.03^ef^	1.68 ± 0.07^b^
B3	7.79 ± 0.68^e^	15.66 ± 1.10^gh^	0.03 ± 0.06	−0.10 ± 0.03	67.11 ± 3.37	0.24 ± 0.02^b^	0.40 ± 0.06^d^	1.01 ± 0.03^fg^
B4	12.76 ± 0.76^b^	37.87 ± 4.22^b^	0.04 ± 0.12	−0.08 ± 0.03	69.92 ± 1.16	0.18 ± 0.01^def^	0.30 ± 0.01^ef^	1.17 ± 0.03^ef^
B5	9.92 ± 0.68^cd^	18.22 ± 0.88^g^	0.04 ± 0.09	−0.02 ± 0.26	67.68 ± 1.66	0.17 ± 0.01^efg^	0.35 ± 0.01^de^	0.93 ± 0.14^g^
P	4.07 ± 0.76^f^	22.83 ± 2.54^f^	0.01 ± 0.06	−0.10 ± 0.05	69.36 ± 1.72	0.13 ± 0.01^h^	0.27 ± 0.04^f^	0.92 ± 0.01^g^
N	15.68 ± 0.73^a^	69.52 ± 2.17^a^	0.07 ± 0.10	−0.03 ± 0.06	70.14 ± 1.36	0.20 ± 0.01^cd^	0.27 ± 0.02^f^	1.64 ± 0.16^bc^

*Note:* Data are showed with means ± SD for three replicates in each group, and the upper lowercase letters represent significantly different (*p* < 0.05).

Abbreviations: ACP, acid phosphatase; AKP, alkaline phosphatase; ALT, alanine aminotransferase; AMS, *α*-amylase; AST, aspartate aminotransferase; FBAs, fermented bile acids; HDL, high-density lipoprotein; LDL, low-density lipoprotein; TG, triglycerides.

**Table 6:** The immune-related factors in white shrimp after treated 60 days.

Groups	AKP	ALF	proPO	*α*2 M
A1	2.23 ± 0.15^b^	1.41 ± 0.30^g^	7.92 ± 0.55^b^	0.10 ± 0.06^d^
A2	0.40 ± 0.06^de^	2.18 ± 0.35^g^	0.05 ± 0.02^d^	0.08 ± 0.01^d^
A3	0.02 ± 0.01^e^	2.21 ± 0.56^g^	0.11 ± 0.03^d^	0.12 ± 0.06^cd^
A4	6.36 ± 0.40^a^	5.20 ± 1.06^f^	0.25 ± 0.05^d^	0.08 ± 0.03^d^
A5	1.06 ± 0.29^c^	11.81 ± 1.58^d^	0.28 ± 0.09^d^	0.08 ± 0.02^d^
B1	0.29 ± 0.08^de^	7.74 ± 1.07^e^	0.43 ± 0.14^cd^	0.34 ± 0.09^a^
B2	2.02 ± 0.51^b^	3.75 ± 0.93^fg^	0.25 ± 0.06^d^	0.13 ± 0.05^cd^
B3	0.08 ± 0.03^e^	10.96 ± 1.03^d^	0.35 ± 0.03^cd^	0.10 ± 0.02^b^
B4	0.62 ± 0.06^d^	2.97 ± 0.71^fg^	0.11 ± 0.04^d^	0.06 ± 0.02^b^
B5	0.12 ± 0.03^e^	23.75 ± 2.03^a^	0.52 ± 0.16^cd^	0.27 ± 0.06^ab^
P	0.34 ± 0.09^de^	15.94 ± 2.83^c^	0.77 ± 0.13^c^	0.22 ± 0.10^bc^
N	0.04 ± 0.01^e^	21.43 ± 1.29^b^	11.69 ± 0.56^a^	0.34 ± 0.09^a^

*Note:* Data are showed with means ± SD for three replicates in each group, and the upper lowercase letters represent significantly different (*p* < 0.05).

Abbreviations: AKP, alkaline phosphatase; ALF, antilipopolysaccharride factor.

## Data Availability

All data generated or analyzed during this study are included in this published article.
